# NIA Division of Behavioral and Social Research Priorities in Health Disparities

**DOI:** 10.1093/geroni/igab046.1404

**Published:** 2021-12-17

**Authors:** Frank Bandiera

**Affiliations:** National Institute on Aging, Bethesda, Maryland, United States

## Abstract

This presentation will include priority research areas in health disparities in the NIA Division of Behavioral and Social Research (BSR). It will include a portfolio analysis and description of BSR program and research in health disparities. Specifically, the 2019 BSR National Advisory Committee on Aging (NACA) recommended BSR’s number one research priority in health disparities. In 2020 BSR held a workshop on structural racism. BSR supports funding announcements in health disparities in Alzheimer’s Disease and Related Dementias, sleep, Native American Health, and immigration among others. Key themes for BSR include racial disparities that often center on context and resources, that is, factors such as residential segregation, rurality, individual and neighborhood SES, and access to health care; persistent racial differences in chronic health conditions; disparities in health systems; immigration and nativity closely linked with race and health outcomes; and racial discrimination linked to poorer mental health and psychological stress.

